# Case Report: Neurolymphomatosis of the sciatic nerve as a presentation of relapsed diffuse large B-cell lymphoma

**DOI:** 10.3389/fonc.2026.1763614

**Published:** 2026-05-07

**Authors:** Jingbo Wang, Yuyu Fu, Jiandi Hu, Mingzhu Zhang, Peng Yu

**Affiliations:** 1 Qingdao University, Qingdao, Shandong, China; 2 Department of Ultrasound Medicine, Yuhuangding Hospital, Yantai, Shandong, China

**Keywords:** diffuse large b-cell lymphoma, neurolymphomatosis, neuropathy, sciatic nerve, ultrasonography, ultrasound-guided core-needle biopsy

## Abstract

**Background:**

Neurolymphomatosis (NL) is an uncommon manifestation of lymphoma caused by direct infiltration of the peripheral nervous system by lymphoma cells. Early recognition is particularly difficult when deep nerves such as the sciatic nerve are involved. Combining contrast-enhanced MRI and PET/CT to assess anatomic extent and metabolic activity, together with nerve ultrasound (US) to identify characteristic signs and guide biopsy, may improve diagnostic accuracy.

**Case presentation:**

A 71-year-old man with systemic diffuse large B-cell lymphoma (DLBCL), histologically confirmed by gastric biopsy, developed progressive left-leg neuropathic pain, numbness, and foot drop during rituximab maintenance after achieving complete remission (CR). Common peroneal nerve decompression yielded only transient pain relief. Nerve US subsequently showed marked thickening of the sciatic nerve and its branches, with a surrounding hypoechoic rim forming the characteristic “fried egg sign,” together with increased perineural echogenicity. MRI showed abnormalities along the sciatic nerve course, and whole-body ^18F-FDG PET/CT demonstrated metabolically active disease along the sciatic nerve course and defined the overall extent of involvement. Ultrasound-guided core-needle biopsy (US-CNB) confirmed NL, and immunohistochemistry demonstrated CD20 positivity, supporting B-cell lymphoma involvement. Given the patient’s age and risk of neurotoxicity, treatment with cyclophosphamide preconditioning followed by glofitamab plus obinutuzumab achieved rapid clinical improvement and partial remission (PR) on imaging. At 3 months, MRI showed interval reduction of the lesion, and follow-up US demonstrated resolution of the hypoechoic rim with decreased diameters of the sciatic, tibial, and common peroneal nerves.

**Conclusions:**

NL should be considered in patients with otherwise unexplained, progressive lower-limb neuropathy and a current or prior history of lymphoma. In this case, nerve US played a central role by demonstrating the characteristic “fried egg sign,” localizing the lesion, and enabling US-CNB for tissue confirmation, while contrast-enhanced MRI and whole-body ^18F-FDG PET/CT provided complementary information on neural involvement and overall disease extent. An ultrasound-centered multimodal approach may help shorten time to diagnosis and facilitate timely treatment and neurological recovery.

## Introduction

Neurolymphomatosis (NL) refers to the direct infiltration of the peripheral nervous system by lymphoma cells, most commonly associated with diffuse large B-cell lymphoma (DLBCL) and typically presenting as painful peripheral neuropathy (1, 2). Because of its non-specific clinical manifestations, particularly when deep nerves are involved, NL is frequently misdiagnosed as compressive or inflammatory peripheral neuropathy. In recent years, multimodal imaging techniques such as PET/CT and ultrasound (US) have become increasingly important for early detection of NL (3, 4). We report a case of systemic DLBCL, histologically confirmed by gastric biopsy, in which NL developed during maintenance therapy, highlighting the value of US in detecting deep nerve involvement. Relevant imaging features, in addition to the diagnostic and treatment course of NL, are also reviewed in conjunction with the current literature.

## Case description

A 71-year-old male patient was diagnosed in January 2024 with systemic diffuse large B-cell lymphoma (DLBCL), germinal center B-cell-like (GCB) subtype, based on gastric biopsy findings. His past medical history was notable for type 2 diabetes mellitus of 11 years’ duration, treated with metformin and glimepiride. He had previously been hospitalized for diffuse large B-cell lymphoma and remained clinically stable after discharge. His history was otherwise unremarkable for major neurologic or cardiovascular disease. He had no known family history of similar disease or hereditary disorders. He was a non-smoker and did not consume alcohol. ^18F-FDG PET/CT showed multi-organ involvement, and the disease was staged as Ann Arbor stage IV with a high International Prognostic Index score. After treatment with an alternating R-CDOP/R-CHOP regimen, the patient achieved complete remission (CR). He subsequently entered maintenance therapy with rituximab and intrathecal methotrexate for central nervous system (CNS) prophylaxis.

Eight months later, the patient developed unexplained numbness, pain, and foot drop involving the left lower leg and dorsum of the foot. Neurologic examination showed left foot drop, with marked weakness graded as Medical Research Council (MRC) grade 0/5 for ankle dorsiflexion, foot eversion, and toe dorsiflexion. Pain sensation was decreased over the dorsolateral aspect of the left foot and the lateral aspect of the left calf, whereas sensation elsewhere was preserved. A Tinel sign was present at the fibular head. Toe flexion was preserved, and distal perfusion was intact. An electrodiagnostic study was also performed. Nerve conduction testing demonstrated slowed motor and sensory conduction velocities in both lower limbs, while bilateral tibial H-reflexes showed prolonged latencies with reduced amplitudes and waveform dispersion, consistent with peripheral neurogenic injury. Needle electromyography was not performed because of hyperglycemia and the chronic course of symptoms. The initial US suggested focal compression of the common peroneal nerve with proximal nerve enlargement. The patient subsequently underwent common peroneal nerve decompression, after which the pain was transiently relieved, but numbness over the dorsum of the foot and foot drop persisted. Over the following two months, swelling of the left lower limb progressively worsened. To further clarify the cause and exclude postoperative complications, US of the lower limbs and soft tissues was performed, revealing no thrombus or mass but only soft tissue edema. Further nerve US revealed marked thickening of the left sciatic nerve and its branches, with a surrounding hypoechoic area forming the characteristic “fried egg sign.” In addition, the perineural soft tissues were visibly thickened with increased echogenicity. MRI revealed abnormal signal changes within the muscle groups along the sciatic nerve pathway. The diagnosis of NL was ultimately confirmed by ultrasound-guided core-needle biopsy (US-CNB). To minimize the risk of direct sciatic fascicular injury, the biopsy targeted the infiltrated epineurial and perineural soft tissue adjacent to the sciatic nerve rather than the nerve fascicles themselves. Histopathologic examination showed diffuse infiltration by atypical lymphoid cells. Immunohistochemistry demonstrated diffuse positivity for CD20, together with positivity for PAX5 and CD79a, supporting B-cell lineage, and the overall findings were consistent with diffuse large B-cell lymphoma involvement.

Considering the patient’s advanced age and risk of neurotoxicity, an individualized chemotherapy regimen was used. Cyclophosphamide was given as preconditioning, followed by a combination of glofitamab and obinutuzumab. After the first cycle, the patient reported improvement in left lower-limb pain and swelling, and MRI demonstrated a reduction in lesion size.

**Figure 1:**
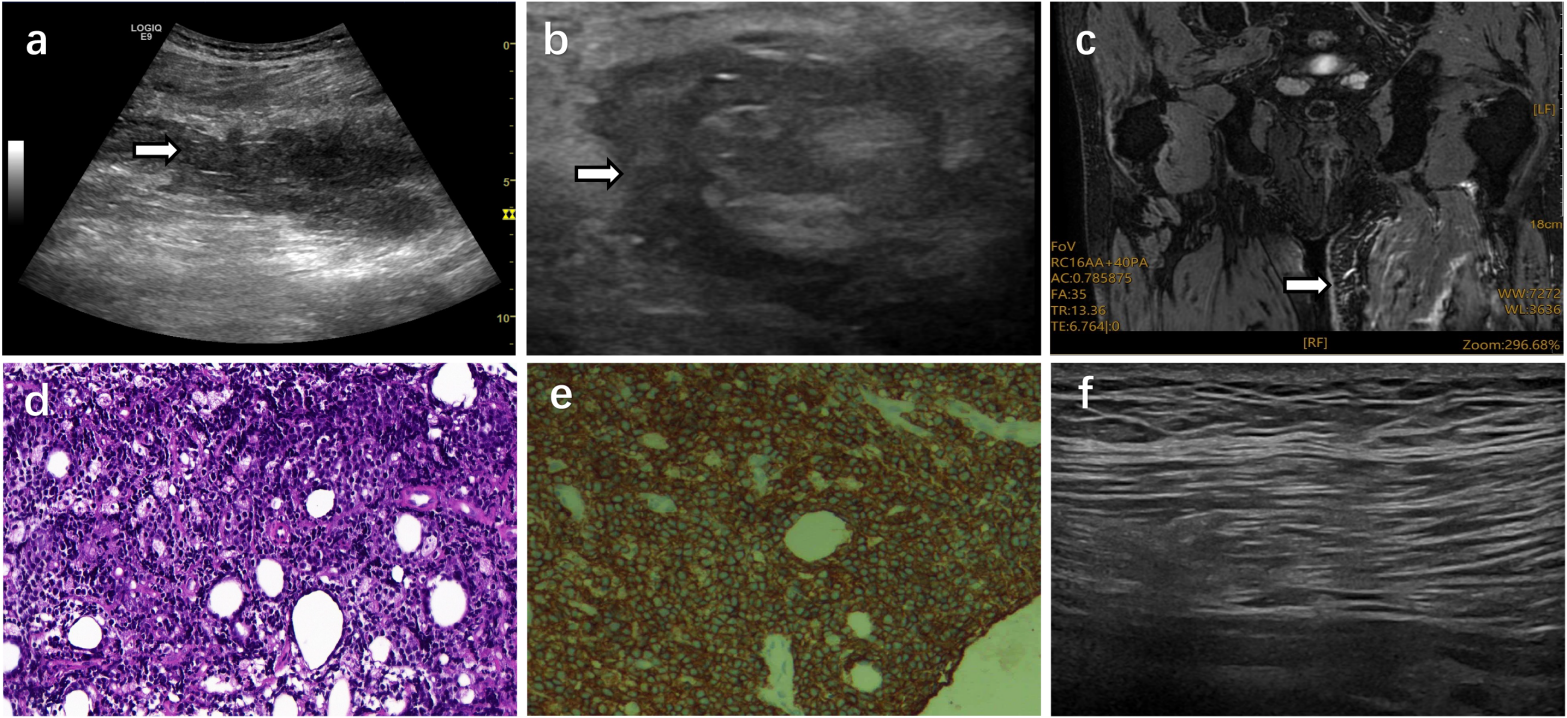
Imaging and histopathological findings of the sciatic nerve and its branches. **(a)** US showed diffuse thickening and heterogeneous echotexture of the left sciatic nerve along its long axis, without evidence of a focal mass, consistent with infiltrative involvement; **(b)** US showed marked thickening of the left sciatic nerve with a surrounding hypoechoic area, forming the characteristic “fried egg sign”; **(c)** MRI (T1-weighted imaging) showed abnormal signal changes within the posterior muscle group of the left thigh, with blurred margins and patchy hyperintensity in the surrounding soft tissues; **(d)** Histopathology (H&E staining, ×400) of the biopsy specimen from the infiltrated epineurial and perineural soft tissue adjacent to the sciatic nerve showed atypical lymphoid infiltration consistent with lymphoma involvement; **(e)** Immunohistochemistry showed diffuse CD20 positivity in the lesional cells, supporting B-cell lymphoma involvement; **(f)** Follow-up US showed that the diameter of the sciatic nerve had decreased compared to previous measurements, and the previously observed “fried egg sign” had resolved.

**Figure 2:**
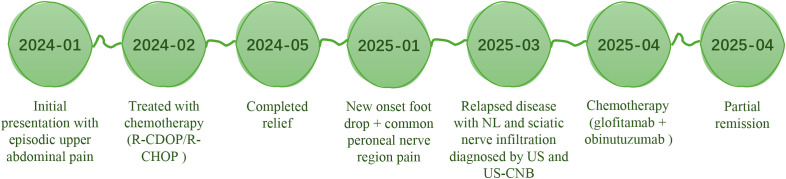
Timeline showing the sequence of events.

Follow-up MRI at three months after treatment showed partial resolution of the original lesion. US showed disappearance of the perineural hypoechoic area surrounding the sciatic nerve, with a marked reduction in the inner diameters of the previously enlarged sciatic, tibial, and common peroneal nerves, indicating disease stability (**Figure 1**). After treatment, pain over the dorsolateral aspect of the left foot and lateral calf resolved, dorsiflexion strength improved from MRC grade 0/5 to 1/5, and numbness over the dorsolateral foot was reduced. Swelling also improved, and the patient remained clinically stable at the latest follow-up (**Figure 2**).

## Discussion

NL denotes lymphomatous infiltration of peripheral nerves, roots, or plexuses and is most frequently associated with DLBCL. Diagnostic delay and initial misclassification as benign neuropathies are frequent because the presentation mimics radiculopathy or entrapment neuropathy and depends on the site involved (2, 5). Baehring et al. reported that over half of NL patients were initially misdiagnosed as benign peripheral neuropathies, and the average diagnosis period was more than three years (1). In our case, although the patient experienced partial pain relief after common peroneal nerve decompression, the swelling symptoms persisted. Follow-up US demonstrated the characteristic “fried egg sign”, and US-guided needle biopsy confirmed NL. This highlights the insidious early presentation of NL and underscores the important value of nerve US in early recognition.

Consistent with prior recommendations, the diagnosis of NL is best supported by a multimodal approach that integrates imaging findings with histopathology. Lee S-H et al. explicitly advocated an MRI–PET/CT–electrodiagnostic triad to expedite confirmation; building on this framework, we additionally highlight the value of US in lesion detection and biopsy guidance (6). Contrast-enhanced MRI and ^18F-FDG PET/CT provide complementary information: MRI helps delineate neural anatomy and structural involvement, whereas PET/CT is valuable for identifying metabolically active disease and assessing whole-body extent. However, neither modality is uniformly diagnostic, and false-negative findings may occur. Accordingly, imaging results should be interpreted in conjunction with clinical presentation, US, and targeted biopsy when feasible (2, 7–10). In addition, US has shown significant value in detecting NL due to its high resolution, dynamic capability, and non-invasive nature, particularly in identifying nerve enlargement, increased intraneural vascularity, and the characteristic “fried egg sign” (11). Lee et al. described this sign as a preserved central hyperechoic nerve fascicle surrounded by a hypoechoic area on transverse view, indicating lymphomatous infiltration (4). In this case, increased echogenicity of the perineural soft tissues provided supplementary evidence of tumor infiltration (12). NL often demonstrates continuous infiltration along the nerve axis, and US allows dynamic, full-length tracking of affected nerves for comprehensive lesion assessment, reducing the risk of missed diagnosis or underestimation of severity. In our case, initial MRI findings were negative, whereas US detected the characteristic “fried egg sign” and successfully guided needle biopsy, highlighting its practical added value in the diagnostic pathway of NL. According to the literature review summarized in **Table 1** (6, 10, 13–25), many sciatic-nerve NL cases were identified by US and ultimately confirmed by US-CNB, supporting US as a practical front-line tool. The value of this report lies in its more complete depiction of an ultrasound-centered diagnostic and follow-up pathway, from initial attribution to a more common peripheral neuropathy process to recognition of characteristic sonographic findings, histologic confirmation by ultrasound-guided core-needle biopsy, and interval follow-up after treatment. In addition, the parallel improvement on follow-up US and MRI further supports the practical role of US not only in lesion detection and biopsy guidance but also in treatment monitoring.

**Table 1:** Chronological summary of 16 published cases in which sciatic nerve neurolymphomatosis was the leading diagnostic feature of lymphoma or the index manifestation at the time of lymphoma recurrence.

Reference first author, year	Age sex	Initial impression	Key radiology	Biopsy method & diagnosis	Treatment	Response
(blinded information), 2025 (present article)	71 M	Unexplained numbness, pain, and foot drop involving the left lower leg and dorsum of the foot	US:Marked thickening of the left sciatic nerve and its branches, with a surrounding hypoechoic area	US-CNB:DLBCL	Chemotherapy	PR after 3 mo of chemotherapy
Soleimani,2023 (11)	69 M	Swelling in the left popliteal fossa, neuropathic pain, and weakness	US: a heterogeneous echogenicity mass with internal vascularity in the popliteal fossa	US-CNB:DLBCL	Chemotherapy	PR at short-term FU
Zhao, 2021 (12)	54 M	Pain and numbness in the right lower limb	PET-CT: Fusiform gluteal soft-tissue mass	Percutaneous needle biopsy: DLBCL	Chemotherapy	PR after 1 cycle of chemotherapy
Kim, 2021 (13)	57 M	Weakness and pain in left leg	MRI: homogeneous fusiform mass at the left greater sciatic foramen	US-CNB:DLBCL	Radiotherapy + chemotherapy	PR after radiotherapy and chemotherapy
Aboueldahab, 2021 (14)	55 M	Worsening pain and weakness in right leg	PET/CT: FDG-avid involvement of the lumbosacral nerve roots	DLBCL	Radiotherapy + chemotherapy	PR after chemotherapy;CR after radiotherapy
Lee, 2020 (6)	64 F	Left lower leg dysfunction, stiffness/numbness of the left calcaneus region, drop foot, and limping gait	US: a heterogeneous echogenicity mass in the left popliteal fossa	US-CNB:DLBCL	Chemotherapy	Brain metastases at 10 mo after first relapse
Bourque, 2019 (15)	67 F	A palpable lump in the distal posterior right thigh	US: a hypoechoic elongated oval-shaped longitudinally oriented mass	US-CNB:DLBCL	Chemotherapy	PR after 5 cycles of chemotherapy
Schuster, 2018 (16)	54 F	Pain and numbness in the left lateral foot and calf, left foot drop and intermittent radiating pain in the left posterior thigh	MRI: Thickening, edema, and enhancement of the left L5–S1 ventral rami and proximal sciatic nerve	CSF flow cytometry: CNS lymphoma	Chemotherapy	N/A
Moussa, 2018 (17)	80 F	Numbness and paresthesia in the right foot	MRI: a lobulated, soft tissue tumor in the pre-Achilles fat planes	US-CNB:DLBCL	Chemotherapy	CR after 3 mo of chemotherapy
Brandstadter, 2015 (18)	64 F	Progressive pain, weakness, and numbness in right leg	MRI: thickening and contrast enhancement of the right S1 nerve root and the right distal sciatic, tibial, and common peroneal nerves	DLBCL	Radiotherapy + chemotherapy	PR after 10 mo of radiotherapy and chemotherapy
Advani, 2015 (19)	72 M	Progressive paresthesia, pain, and weakness of the left plantar foot, with extension to the posterior thigh and calf	MRI: A heterogeneously enhancing fusiform elongated mass intimately associated with the distal sciatic nerve	Surgical biopsy: DLBCL	Chemotherapy	CR after 6 cycles of chemotherapy
Strobel, 2007 (20)	59 M	Fluctuating gluteal pain and progressive palsy of the left lower extremity	FDG PET/CT: a high FDG uptake of the left sciatic nerve	DLBCL	Chemotherapy	CR after 6 cycles of chemotherapy
Descamps, 2006 (21)	55 M	weakness and pain in left foot	MRI: abnormal sciatic trunk	Surgical biopsy: DLBCL	Chemotherapy	Remission at 4 mo; sustained remission at 48 mo
Quiñones-Hinojosa, 2000 (22)	52 M	Progressive weakness and sensory disturbance of the right leg	MRI: fusiform lesion in sciatic nerve	Surgical biopsy:Burkitt-like HGBL	Chemotherapy	Died of respiratory failure after 4 mo of chemotherapy
Kanamori, 1995 (23)	34 M	Muscle weakness and dysesthesia in the left leg	MRI and gallium scintigraphy	Surgical biopsy: T-cell NHL	Radiotherapy + chemotherapy	PR; NED at 30 mo posttreatment
Purohit, 1986 (24)	64 F	Progressive leg weakness and sensory loss	CT thigh: enlarged distal sciatic segment	Surgical biopsy:B-cell lymphoma	Surgical resection	No evidence of lymphoma elsewhere at 9 mo

US, Ultrasound; US-CNB, Ultrasound-guided core-needle biopsy; Surgical biopsy, open incisional or excisional biopsy; CT, Computed tomography; MRI, Magnetic resonance imaging; PET/CT, Positron-emission tomography/CT; CSF, Cerebrospinal fluid; CNS, Central nervous system; DLBCL, Diffuse large B-cell lymphoma; HGBL, High-grade B-cell lymphoma; NHL, Non-Hodgkin lymphoma; CTCL, Cutaneous T-cell lymphoma; NED, No evidence of disease; CR, Complete remission; PR, Partial remission; SD, Stable disease; PD, Progressive disease; FU, Follow-up; mo, months; post-treatment, measured from the end of initial therapy; cycle(s), number of chemotherapy cycles; N/A, not available.

Histopathological examination remains the gold standard for diagnosing NL. When deep location or comorbidity limits open biopsy, US-CNB offers a minimally invasive alternative that still enables immunophenotyping and molecular subtyping to guide individualized therapy; as summarized in **Table 1**, numerous cases adopted this strategy with satisfactory diagnostic yield. In our case, the patient was confirmed as the non-GCB subtype of DLBCL, which is relatively common in NL and has been associated with poorer prognosis (20, 26). Although DLBCL is the predominant histologic type associated with NL, other lymphoma subtypes have also been reported, including low-grade B-cell lymphoma, Burkitt lymphoma, peripheral T-cell lymphoma, and, more rarely, NK/T-cell lymphoma. Overall, however, the majority of reported NL cases still arise in the setting of B-cell non-Hodgkin lymphoma, particularly DLBCL (13, 27, 28). In clinical practice, approximately half of patients cannot undergo confirmatory needle biopsy due to deep location of the lesions or poor general condition, leaving clinicians to rely on symptoms and imaging for a presumptive diagnosis followed by empirical treatment (29, 30).

Currently, there is no standardized treatment guideline for NL. In clinical practice, treatment strategies are largely adapted from those used for DLBCL and primary CNS lymphoma. High-dose methotrexate combined with rituximab remains the mainstay. Since NL often involves multiple peripheral nerves sites, radiotherapy is generally reserved for controlling localized refractory neuropathic pain or residual disease. In recent years, antibody-based therapies such as glofitamab have shown encouraging efficacy in elderly patients and in those with relapsed or refractory NL (31). As summarized in **Table 1**, case-based evidence shows that partial remission (PR) or CR can be achieved after chemo-radiotherapy in sciatic-nerve NL. Nevertheless, the overall prognosis of NL is generally poor. Grisariu et al. reported a median survival of only 10 months and a 3-year survival rate of 24%, with an even worse prognosis in patients with concomitant CNS lymphoma (2, 32). A 2024 BJC Reports synthesis of 559 neurolymphomatosis cases found a median overall survival of 12 months and highlighted that diagnostic delays remain common, underscoring the need for faster recognition and timely tissue confirmation (33). In our case, the patient achieved neurological improvement and PR after an individualized chemotherapy regimen without anthracyclines or corticosteroids, suggesting that elderly patients may still benefit from timely and appropriate intervention.

Neurological recovery in NL is closely related to treatment timing. Some cases present as nerve mass-like lesions and are misdiagnosed as benign lesions such as schwannomas. Gobinath et al. reported a patient misdiagnosed preoperatively as a schwannoma, with postoperative pathology finally confirming DLBCL, leading to a delay in systemic treatment (34). Therefore, for patients with unexplained, progressive peripheral neuropathy, especially sciatic neuropathy, and a current or prior lymphoma history, NL should be considered in the differential diagnosis. A rapid evaluation using multiple modalities is warranted. Contrast-enhanced MRI and whole-body PET/CT help detect disease, define the extent of neural involvement, and assess metabolic activity, whereas US can localize the lesion precisely, characterize sonographic abnormalities, and identify an appropriate sampling target. When feasible, we use US-CNB to obtain tissue for histopathology and biomarker testing.

## Conclusion

We report a case of NL presenting as unilateral sciatic neuropathy, in which nerve US played a central role by demonstrating the characteristic “fried egg sign,” localizing the lesion, and enabling US-CNB for tissue confirmation. PET/CT and contrast-enhanced MRI provided complementary information on neural involvement, disease extent, and metabolic activity. In patients with otherwise unexplained, progressive lower-limb neuropathy and a current or prior history of lymphoma, NL should be included in the differential diagnosis. A practical multimodal approach centered on nerve US, with MRI and PET/CT used to further assess neural involvement and overall disease extent, may help shorten time to diagnosis and facilitate timely treatment. As a rapid, accessible, and low-risk tool, US may also support follow-up assessment and contribute to better neurological recovery through earlier tissue confirmation and intervention.

## Data Availability

The original contributions presented in the study are included in the article/supplementary material. Further inquiries can be directed to the corresponding author.
